# Preparation of Two New Diasteromeric Chiral Stationary Phases Based on (+)-(18-Crown-6)-2,3,11,12-tetracarboxylic Acid and (*R*)- or (*S*)-1-(1-Naphthyl)ethylamine and Chiral Tethering Group Effect on the Chiral Recognition

**DOI:** 10.3390/molecules21081051

**Published:** 2016-08-12

**Authors:** Rajalingam Agneeswari, Ji Yeong Sung, Eun Sol Jo, Hee Young Jeon, Vellaiappillai Tamilavan, Myung Ho Hyun

**Affiliations:** Department of Chemistry and Chemistry Institute for Functional Materials, Pusan National University, Pusan 609-735, Korea; agneeswarichem@gmail.com (R.A.); jysung@kbsi.re.kr (J.Y.S.); dmsthf4834@hanmail.net (E.S.J.); heeyoung0906@naver.com (H.Y.J.); tamilavan4811@gmail.com (V.T.)

**Keywords:** chiral stationary phase, chiral tethering group, enantiomer separation, liquid chromatography

## Abstract

Two new diastereomeric chiral stationary phases (CSPs) based on (+)-(18-crown-6)-2,3,11,12-tetracarboxylic acid as a chiral tethering group and a Π-basic chiral unit such as (*R*)-1-(1-naphthyl)ethylamine (CSP **1**) or (*S*)-1-(1-naphthyl)ethylamine (CSP **2**) were prepared. The two CSPs were applied to the enantiomeric separation of *N*-(3,5-dinitrobenzoyl)-1-phenylalkylamines and *N*-(3,5-dinitrobenzoyl)-α-amino acid derivatives using 20% isopropyl alcohol in hexane as a normal mobile phase. To elucidate the effect of the two chiral units on the chiral recognition, the chiral recognition abilities of the two CSPs were compared with each other and with that of a CSP (CSP **3**) based on (*R*)-1-(1-naphthyl)ethylamine. From the chromatographic chiral recognition results, (*R*)-1-(1-naphthyl)ethylamine and (+)−(18-crown-6)-2,3,11,12-tetracarboxylic acid constituting CSP **1** were concluded to show a cooperative (“matched”) effect on the chiral recognition while (*S*)-1-(1-naphthyl)ethylamine and (+)-(18-crown-6)-2,3,11,12-tetracarboxylic acid constituting CSP **2** were concluded to show an uncooperative (“mismatched”) effect on the chiral recognition. From these results, it was concluded that (+)-(18-crown-6)-2,3,11,12-tetracarboxylic acid can be successfully used as a chiral tethering group for the preparation of new CSPs.

## 1. Introduction

Separating enantiomers on liquid chromatographic chiral stationary phases (CSPs) has been known to be quite effective as an analytical method for the determination of the enantiomeric composition of chiral compounds and as a preparative method for the separation of enantiomers [1,2,3,4,5]. During the last several decades, quite effective CSPs have been developed by utilizing various chiral selectors. For example, polysaccharide derivatives [6,7], cyclodextrins [8], macrocyclic antibiotics [9,10], cyclofructans [11,12], Π-acidic or Π-basic aromatic chiral compounds [13,14], cinchona alkaloids [15,16], chiral ligand exchange materials [17,18] and chiral crown ethers [19,20,21] have been widely used as chiral selectors for the development of CSPs. Those chiral selectors are based on an assemblage of repeating or non-repeating chiral subunits playing together for chiral recognition or based on a single chiral unit. In addition, diastereomeric chiral selectors consisting of two chiral units have been reported to show the “matched” effect on the chiral recognition when the two chiral units are cooperative and, consequently, enhance the chiral recognition or the “mismatched” effect on the chiral recognition when the two chiral units are uncooperative and, consequently, diminish the chiral recognition, according to the stereochemistry of the two chiral units. One of the two chiral units has usually been used as a chiral tethering group when the diastereomeric chiral selectors were bonded to silica gel. Even though the term of the “matched/mismatched” effect has been originally used to rationalize the different ratios of two diastereomers synthesized in the reaction of an enantiomerically pure substrate with an enantiomerically pure reagent [22], the concept of the “matched/mismatched” effect has also been successfully used to rationalize the chiral recognition behaviors of diastereomeric CSPs. For example, when the two diastereomeric Π-basic aromatic chiral compounds consisting of (*R*)- or (*S*)-1-(1-naphthyl)ethylamine and (*S*)-naproxen were bonded to silica gel through the (*S*)-naproxen tethering group, the two chiral units were found to show the “matched” or “mismatched” effect on the chiral recognition according to the stereochemistry of the 1-(1-naphthyl)ethylamine unit [23]. Similarly, when diastereomeric chiral crown ethers incorporating (*R*)-3,3’-diphenyl-1,1’-binaphthyl and (*R*,*R*)- or (*S*,*S*)-tartaric acid unit as chiral barriers were bonded to silica gel through the (*R*,*R*)- or (*S*,*S*)-tartaric acid unit as a chiral tethering group, the resulting two diastereomeric CSPs were also found to show the “matched” or “mismatched” effect on the chiral recognition according to the stereochemistry of the tartaric acid unit [24,25]. However, CSPs based on diastereomers consisting of a Π-basic aromatic chiral unit and chiral crown ether have not been reported yet.

**Figure 1:**
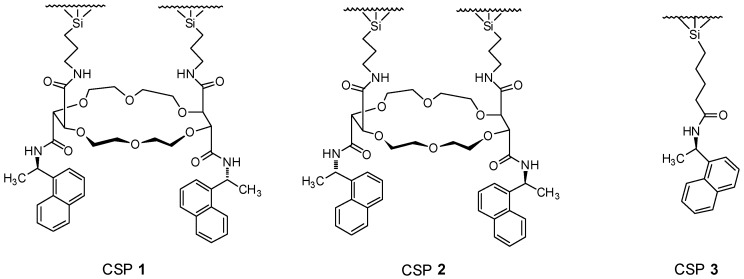
Structures of CSP **1**, CSP **2** and CSP **3**.

In this study, two new diastereomeric CSPs (CSP **1** and CSP **2**, Figure 1) based on two chiral units such as (*R*)- or (*S*)-1-(1-naphthyl)ethylamine as a Π-basic aromatic chiral unit and (+)-(18-crown-6)-2,3,11,12-tetracarboxylic acid as a chiral tethering group were prepared and applied to the enantiomeric separation of some racemic chiral compounds under the normal mobile phase condition. The comparison of the chromatographic chiral recognition results of the two CSPs with each other and with those of the CSP based on (*R*)-1-(1-naphthyl)ethylamine as a single chiral unit (CSP **3**, Figure 1) is expected to elucidate the “matched” or “mismatched” effect of the two chiral units consisting of the two new diastereomeric CSPs on the chiral recognition. Through this study, we hope to demonstrate that (+)-(18-crown-6)-2,3,11,12-tetracarboxylic acid can be used as a useful chiral tethering group for the preparation of effective CSPs.

## 2. Results and Discussion

**Scheme 1:**
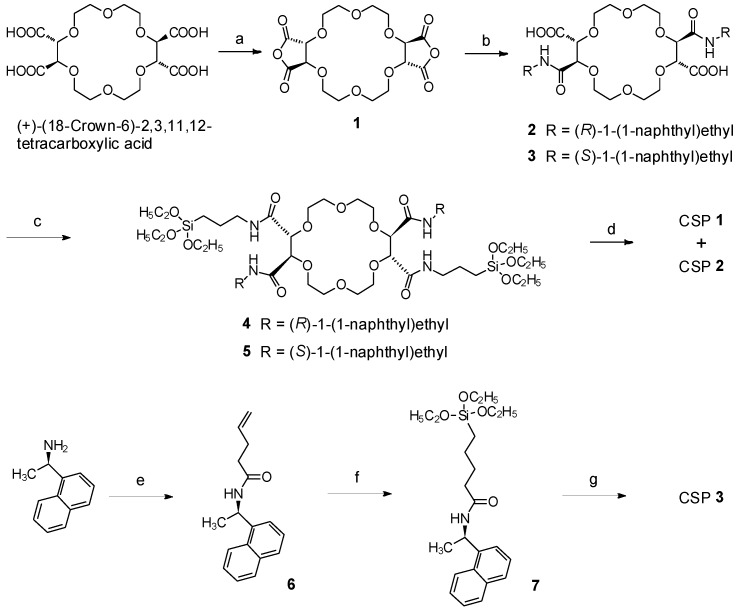
Preparation of CSP **1**, CSP **2** and CSP **3**. (**a**) Acetyl chloride, reflux; (**b**) (*R*)-1-(1-naphthyl)ethylamine or (*S*)-1-(1-naphthyl)ethylamine, triethylamine, methylene chloride. 0 °C to reflux; (**c**) 3-aminopropyltriethoxysilane, 2-ethoxy-1-ethoxycarbonyl-1,2-dihydroquinoline (EEDQ), methylene chloride, room temperature; (**d**) 5 µm silica gel, toluene, Dean-Stark trap; (**e**) 4-petenoyl chloride, triethylamine, methylene chloride, 0 °C; (**f**) triethoxysilane, H_2_PtCl_6_·6H_2_O and (**g**) 5 µm silica gel, toluene, Dean-Stark trap.

Previously, (+)-(18-crown-6)-2,3,11,12-tetracarboxylic acid has been used as a very successful chiral selector for the preparation of chiral crown ether–based CSPs [19,20,21]. However, (+)-(18-crown-6)-2,3,11,12-tetracarboxylic acid has not been used as a chiral tethering group for the preparation of diastereomeric CSPs. In this study, (+)-(18-crown-6)-2,3,11,12-tetracarboxylic acid was used as a chiral tethering group for the preparation of diastereomeric CSPs such as CSP **1** and CSP **2**. Scheme 1 shows how (+)-(18-crown-6)-2,3,11,12-tetracarboxylic acid was used as a chiral tethering group for the preparation of CSP **1** and CSP **2**. In addition, Scheme 1 shows the synthetic procedure for the preparation of CSP **3**. To prepare CSP **1** and CSP **2**, (+)-(18-crown-6)-2,3,11,12-tetracarboxylic acid was converted to its dianhydride, **1**, by treating it with acetyl chloride via the known procedure [26]. Then the dianhydride, **1**, was treated with (*R*)-1-(1-naphthyl)ethylamine or (*S*)-1-(1-naphthyl)ethylamine in the presence of triethylamine in methylene chloride to afford *syn*-diamide compound **2** or **3**. The *syn*-diamide structure of compound **2** or **3** is believed to stem from the face-selective *syn*-opening of the dianhydride in the presence of triethylamine [27,28]. Compound **2** or **3**, then, was treated with 3-aminopropyltriethoxysilane in the presence of a coupling agent, 2-ethoxy-1-ethoxycarbonyl-1,2-dihydroquinoline (EEDQ), in methylene chloride to afford triethoxysilyl group–containing compound **4** or **5**. Finally, compound **4** or **5** was bonded to silica gel to afford CSP **1** or CSP **2**. CSP **3** was prepared starting from (*R*)-1-(1-naphthyl)ethylamine. The (*R*)-1-(1-naphthyl)ethylamine was treated with 4-pentenoyl chloride in the presence of triethylamine in methylene chloride at 0 °C to afford terminal double bond–containing amide compound **6**. Then amide compound **6** was hydrosilylated by treating with triethoxysilane and chloroplatinic acid hexahydrate. The resulting triethoxysilyl group–containing compound **7** was bonded to silica gel to afford CSP **3**.

**Figure 2:**
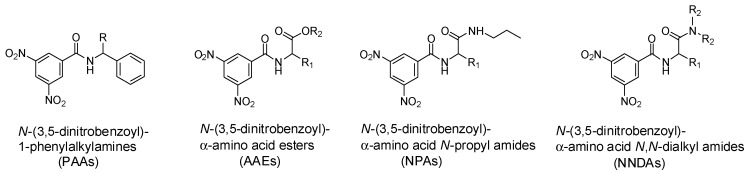
Racemic compounds resolved on CSP **1** and CSP **2**.

CSP **1** and CSP **2** containing Π-basic naphthyl groups were applied to the resolution of Π-acidic *N*-3,5-dinitrobenzoyl derivatives of 1-phenylalkylamines (PAAs), α-amino acid esters (AAEs), α-amino acid *N*-propyl amides (NPAs), and α-amino acid *N*,*N*-dialkyl amides (NNDAs), shown in Figure 2, and the resolution results of the two CSPs were compared with each other.

Table 1 shows the chromatographic results for the resolution of PAAs on CSP **1** and CSP **2** using 20% isopropyl alcohol in hexane as a mobile phase. The two enantiomers were resolved quite well on the two CSPs, but CSP **1** was found to show better chiral recognition than CSP **2**. When the length of the alkyl group at the chiral center was increased from methyl to pentyl, the retention factors and the separation factors decreased on both CSP **1** and CSP **2**. When the length of the alkyl group at the chiral center was increased further, the retention factors decreased continuously, but the separation factors decreased only slightly or remained almost constant. In the case of the enantiomeric separation of PAA-**1**, the elution order was determined by injecting enantiomerically known samples prepared from (*R*)-1-phenylethylamine, but the elution orders for the enantiomeric separation of other analytes were not determined because enantiomerically known samples were not available.

The chromatographic results for the enantiomeric separation of AAEs shown in Figure 2 are summarized in Table 2. The elution orders indicated in Table 2 were determined by injecting enantiomerically known samples. In every case, the (*S*)-enantiomer was eluted first on CSP **1**, whereas the (*R*)-enantiomer was eluted first on CSP **2**. These results indicate that the chiral recognition is determined mainly by the 1-(1-naphthyl)ethylamine component of the CSPs. In the enantiomeric separation of AAEs, CSP **1** was always better (higher α) than CSP **2**, as in the enantiomeric separation of PAAs.

Interestingly, the separation factors (α) increased as the alkyl chain length of the ester O-alkyl group of analytes was increased. In the case of AAE-**5**~AAE **8**, the separation factors (α) increased continuously as the ester alkyl chain length was increased from methyl to n-octyl. When the alkyl chain length of the ester O-alkyl group of analytes was increased, the retention factors (*k*_1_) decreased as usual. The large retention factors (*k*_1_) for the resolution of AAE-**5** and AAE-**9** compared to those for the resolution of AAE-**1** and AAE-**3** might be rationalized by the non-enantioselective Π-Π interaction between the 1-naphthyl group of the stationary phase and the phenyl or benzyl group of analytes. In the presence of a non-enantioselective Π-Π interaction between the stationary phase and analytes, the retention times of the analytes might increase, but the separation factors might decrease. The retention factor (*k*_1_) for AAE-**11** was quite large compared to those of other analytes. The non-enantioselective hydrogen bonding interaction between the 4-hydroxy group of the analyte and the CSP might be the reason for the large retention factor.

The enantiomeric separation of NPAs shown in Figure 2 on CSP **1** and CSP **2** was summarized in Table 3. The elution orders for the enantiomeric separation of NPAs on CSP **1** and CSP **2** were identical to those for the enantiomeric separation of AAEs on CSP **1** and CSP **2**. CSP **1** was also found to be better than CSP **2** in the enantiomeric separation of NPAs. The retention factor (*k*_1_) for the enantiomeric separation of NPA-**6** was also quite large compared to those of other analytes.

NNDAs shown in Figure 2 were also resolved on CSP **1** and CSP **2**. The resolution results are summarized in Table 4. CSP **1** was also found to be better than CSP **2** in the enantiomeric separation of NNDAs. The elution orders were consistent with those for the enantiomeric separation of AAEs and NPAs. In general, the enantiomeric separation of NNDAs on CSP **1** and CSP **2** was not much different from that of corresponding NPAs in terms of the separation factors (α). However, the retention factors (*k*_1_) for the enantiomeric separation of NNDAs on CSP **1** and CSP **2** were always lower than those for the enantiomeric separation of corresponding NPAs. These results indicate that the N-propyl amide N-H hydrogen of NPAs is not involved in the chiral recognition. Instead, the N-propyl amide N-H hydrogen of NPAs is expected to be involved in the non-enantioselective hydrogen bonding interaction with the stationary phase.

**Figure 3:**
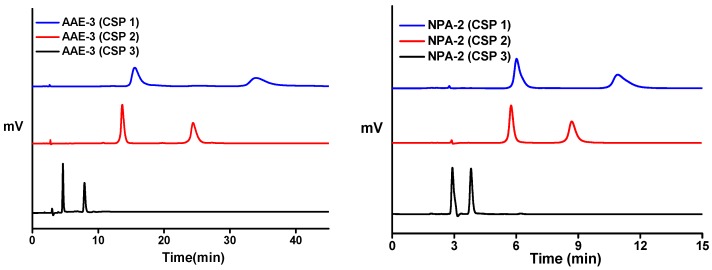
Comparison of the representative chromatograms for the enantiomeric separation of AAE-**3** and NPA-**2** on CSP **1**, CSP **2** and CSP **3**. For the chromatographic conditions, see the footnotes to Table 2 and Table 3.

To investigate the effect of a chiral tethering group such as (+)-(18-crown-6)-2,3,11,12-tetra-carboxylic acid of CSP **1** and CSP **2** on the chiral recognition, the chromatographic results for the enantiomeric separation of some selected analytes on the two CSPs were compared with those on CSP **3** as shown in Table 5. As an example, a comparison of the chromatograms for the enantiomeric separation of AAE-**3** and NPA-**2** on CSP **1**, CSP **2** and CSP **3** is presented in Figure 3. The elution orders on CSP **3** were identical to those on CSP **1**. These results also indicate that the chiral recognition is determined mainly by the 1-(1-naphthyl)ethylamine component of the CSPs. As shown in Table 5, the separation factors (α) on CSP **1** were always greater than those on CSP **3**, except for the enantiomeric separation of NNDA-**11,** but those on CSP **2** were always lower than those on CSP **3**. Even though the differences in the separation factors (α) are not so great, the effects of the second chiral unit of CSP **1** and CSP **2** on the chiral recognition are opposite. From these results, it is concluded that the chiral tethering unit of (+)-(18-crown-6)-2,3,11,12-tetracarboxylic acid of CSP **1** exerts the cooperative (“matched”) effect with the 1-(1-naphthyl)ethylamine unit on the chiral recognition, but the chiral tethering unit of CSP **2** exerts the uncooperative (“mismatched”) effect on the chiral recognition. Interestingly, the retention factors (*k*_1_) on CSP **3** are quite small compared to those on CSP **1** and CSP **2**. CSP **1** and CSP **2** contain many additional non-enantioselective hydrogen bonding sites such as crown ether ring oxygens and additional amide sites compared to CSP **3**. These non-enantioselective hydrogen bonding sites on CSP **1** and CSP **2** can possibly improve the retention of analytes. The density of the 1-(1-naphthyl)ethylamine component of CSP **1** and CSP **2** calculated from the elemental analysis was 0.34 and 0.32 mmol/g, respectively, and these values are higher than that (0.25 mmol/g) of CSP **3**. The higher density of the 1-(1-naphthyl)ethylamine component of the CSPs can also enhance the retention of analytes, but does not seem to always improve the enantioselectivity, as evidenced by the lower separation factors (α) on CSP **2** compared to those on CSP **3**.

## 3. Materials and Methods

### 3.1. Materials and Instruments

All necessary reagents and solvents were available from Sigma-Aldrich (St. Louis, MO, USA) or TCI chemicals and the reagents (Tokyo, Japan) were used without further purification and the solvents were handled in a moisture-free atmosphere. (+)-(18-Crown-6)-2,3,11,12-tetracarboxylic acid was obtained from RS tech (Daejeon, Korea). The purification of compounds was performed by using column chromatography (silica gel, Merck Kieselgel 60, 70–230 mesh ASTM). The nuclear magnetic resonance (NMR) spectra of the compounds were recorded with Varian Mercury Plus spectrometer (300 MHz). Liquid chromatography was performed with an HPLC system consisting of a Waters model 515 HPLC pump (Milford, MA, USA), a Rheodyne model 7725i injector (Rohnert Park, CA, USA) with a 20 μL sample loop, a YoungLin M720 absorbance UV detector (variable wavelength, Seoul, Korea) and a YoungLin Autochro data module (Software: YoungLin Autochro-WIN 2.0 plus). The temperature of the chiral column was controlled by using a Julabo F30 Ultratemp 2000 cooling circulator (Seelbach, Germany). Racemic and optically active analytes were available from a prior study or prepared using the similar reported procedure [29,30]. Injection samples were prepared by dissolving each of racemic and optically active samples in methanol (usually 2.5 mg/mL). The usual injection volume was 5.0 µL.

### 3.2. Synthesis of CSPs

*Synthesis of compound* **2**: The stirred solution of compound **1** (0.85 g, 2.10 mmol), which was prepared using the reported procedure [26,31], in methylene chloride (50 mL) was cooled to 0 °C under an argon atmosphere. A solution of (*R*)-1-(1-naphthyl)ethylamine (0.86 g, 5.00 mmol) and triethylamine (0.70 mL, 5.00 mmol) in methylene chloride (20 mL) was added drop by drop. The reaction mixture was slowly warmed to room temperature and then refluxed for 16 h. Then, the reaction mixture was cooled to room temperature and washed twice with 2 N HCl. The organic layer was dried over Na_2_SO_4_. The solvent was removed under reduced pressure and solid was washed with hexane to afford compound **2** as an off-white solid. Yield: 1.46 g (91%). ^1^H-NMR (300 MHz, CDCl_3_, δ): 1.40–1.60 (m, 6H), 2.80–4.00 (m, 20H), 5.90–6.10 (m, 2H), 7.30–8.10 (m, 14H).

*Synthesis of compound* **3**: Compound **3** was prepared by using the similar procedure for the synthesis of compound **2**. In this reaction, (*S*)-1-(1-naphthyl)ethylamine (0.86 g, 5.00 mmol) was used instead of (*R*)-(+)-1-(1-naphthyl)ethylamine. Yield: 1.40 g (87%). ^1^H-NMR (300 MHz, CDCl_3_, δ): 1.40–1.60 (m, 6H), 2.80–4.00 (m, 20H), 5.90–6.10 (m, 2H), 7.30–8.10 (m, 14H).

*Synthesis of compound* **4**: To a stirred solution of compound **2** (1.40 g, 1.90 mmol) in methylene chloride (50 mL) was added 2-ethoxy-1-ethoxycarbonyl-1,2-dihydroquinoline (EEDQ, 1.90 g, 7.50 mmol) under an argon atmosphere. After 15 minutes, (3-aminopropyl)triethoxysilane (1.75 mL, 7.50 mmol) was added and the reaction mixture was stirred for overnight. Then, the solvent was removed under reduced pressure and the crude material was purified by fast column chromatography (silica gel, methylene chloride:methanol, 90/10, *v*/*v*) to afford light brown sticky mass as compound **4**. Yield: 0.90 g (41.7%). ^1^H-NMR (300 MHz, CDCl_3_, δ): 0.50–0.70 (m, 4H), 1.18(q, 18H), 1.54–1.76 (m, 14H), 2.60–4.00 (m, 28H), 4.40 (s, 4H), 5.90–6.10 (m, 2H), 7.30–8.00 (m, 14H).

*Synthesis of compound* **5**: Compound **5** was prepared using the similar procedure for the synthesis of compound **4**. In this reaction, compound **3** (1.40 g, 1.90 mmol), EEDQ (1.90 g, 7.50 mmol) and (3-aminopropyl)triethoxysilane (1.75 mL, 7.50 mmol) were used. Yield: 0.90 g (41.7%). ^1^H-NMR (300 MHz, CDCl_3_, δ): 0.50–0.70 (m, 4H), 1.18(q, 18H), 1.54–1.76 (m, 14H), 2.60–4.00 (m, 28H), 4.40 (s, 4H), 5.90–6.10 (m, 2H), 7.30–8.00 (m, 14H).

*Synthesis of CSP* **1** *and column packing:* A 250 mL two neck flask equipped with a Dean-Stark trap, a condenser, and an overhead stirrer was charged with silica gel (2.5 g, kromasil, 5 µ) and toluene (150 mL). The mixture was heated to reflux to remove water azeotropically. To the slowly stirred mixture, a solution of compound **4** (0.90 g, 0.80 mmol) in 10mL toluene was added in one portion. The whole mixture was refluxed for 72 h and filtered, washed successively with methanol, acetone, ethyl acetate, methylene chloride, hexane and diethyl ether. Finally, the modified silica gel was dried under vacuum to afford CSP **1** (2.95 g). The loading level of the chiral selector (compound **4**) on silica gel was calculated to be 0.17 mmol/g based on carbon from the elemental analysis (C, 10.24; H, 1.62; N, 0.60). The modified silica gel was slurred in methanol and packed into 150 × 4.6 mm stainless steel HPLC column by using conventional slurry packing method with an Alltech slurry packer.

*Synthesis of CSP* **2** *and column packing:* A similar procedure mentioned for the preparation of CSP **1** was used for the synthesis and column packing for CSP **2**. In this case, compound **5** (0.90 g, 0.78 mmol) was used instead of compound **4**. All other procedures were same. The loading level of the chiral selector (compound **5**) on silica gel was calculated to be 0.16 mmol based on the carbon content from the elemental analysis (C, 10.00; H, 1.74; N, 0.59). The modified silica gel was slurred in methanol and packed into 150 × 4.6 mm stainless steel HPLC column by using conventional slurry packing method with an Alltech slurry packer.

*Synthesis of compound* **6**: The stirred solution of (*R*)-1-(1-naphthyl)ethylamine (1.5 g, 8.76 mmol) in methylene chloride (50 mL) was cooled to 0 °C and triethylamine (1.34 g, 9.63 mmol) was added and then the whole mixture was stirred for 15 min. Then, 4-pentenoyl chloride (1.06 mL, 9.63 mmol) was added drop by drop and stirred for 30 min. The reaction mixture was washed with sodium bicarbonate (10%) solution, 1 N HCl and brine. The organic layer was dried over Na_2_SO_4_. The solvent was removed under reduced pressure and solid was washed with hexane to afford compound **6** as a white solid. Yield: 1.8 g (81%). ^1^H-NMR (300 MHz, CDCl_3_, δ): 1.67 (d, 3H), 2.24 (t, 2H), 2.38 (q, 2H), 4.98 (t, 2H), 5.52–5.58 (m, 2H), 5.58–6.00 (m, 1H), 7.48–7.52 (m, 4H), 7.79 (d, 1H), 7.86 (d, 1H), 8.08 (d, 1H).

*Synthesis of compound* **7**: Compound **6** (0.80 g, 3.16 mmol) and triethoxysilane (30 mL) were taken in a flame dried round bottom flask (100 mL) under an argon atmosphere. The mixture was heated to 80 °C with gentle stirring. To the clear solution, H_2_PtCl_6_·6H_2_O (5 mg in 1 mL isopropyl alcohol) was added drop by drop and the stirring was continued for 2 h. The reaction mixture was cooled to room temperature and concentrated under reduced pressure. The sticky mass was directly subjected to fast column chromatography (silica gel, methylene chloride:methanol, 90/10, *v*/*v*) to afford light brown sticky mass. Yield: 0.8 g (60%). ^1^H-NMR (300 MHz, CDCl_3_, δ): 0.62 (t, 2H), 1.19 (t, 9H), 1.35–1.50 (m, 2H), 1.52–1.54 (m, 5H), 2.15 (t, 2H), 3.77 (q, 6H), 5.66 (d, 1H), 5.88–6.00 (m, 1H), 7.40–7.60 (m, 4H), 7.79 (d, 1H), 7.86 (d, 1H), 8.08 (d, 1H).

*Synthesis of CSP* **3** *and column packing:* A similar procedure described for the preparation of CSP **1** was used for the synthesis and column packing for CSP **3**. In this case, compound **7** (2.7 g, 1.92 mmol) was used. All other procedures were same. The loading level of the chiral selector (compound **7**) on silica gel was calculated to be 0.25 mmol/g based on the carbon content from the elemental analysis (C, 5.73; H, 1.41; N, 0.22). The modified silica gel was slurred in methanol and packed into 150 × 4.6 mm stainless steel HPLC column by using conventional slurry packing method with an Alltech slurry packer.

## 4. Conclusions

The two chiral units consisting of the two new diastereomeric chiral stationary phases (CSPs) based on (+)-(18-crown-6)-2,3,11,12-tetracarboxylic acid as a chiral tethering group and a Π-basic chiral unit such as (*R*)-1-(1-naphthyl)ethylamine (CSP **1**) or (*S*)-1-(1-naphthyl)ethylamine (CSP **2**) were demonstrated to show the “matched” or “mismatched” effect on the chiral recognition, depending on the stereochemistry of the Π-basic chiral unit. For the enantiomeric separation of *N*-(3,5-dinitrobenzoyl)-1-phenylalkylamines and *N*-(3,5-dinitrobenzoyl)-α-amino acid derivatives on the two CSPs, the Π-basic chiral unit was found to play a significant role as a major chiral selector in the chiral recognition, but the tethering (+)-(18-crown-6)-2,3,11,12-tetracarboxylic acid group was found to play an additional role showing the “matched” or “mismatched” effect on the chiral recognition. From these results it was concluded that (+)-(18-crown-6)-2,3,11,12-tetracarboxylic acid can be successfully used as a chiral tethering group in the preparation of new effective CSPs.

## Figures and Tables

**Table 1:** Resolution of PAAs shown in Figure 2 on CSP **1** and CSP **2** ^1^.

PAAs	R	CSP 1		CSP 2	
		*k*_1_	α	*k*_1_	α
PAA-**1**	CH_3_	15.98 (S)	2.19	13.78 (R)	1.95
PAA-**2**	*n*-C_5_H_6_	12.69	1.94	12.01	1.75
PAA-**3**	*n*-C_10_H_21_	9.90	1.88	9.66	1.66
PAA-**4**	*n*-C_13_H_27_	8.34	1.86	8.93	1.66
PAA-**5**	*n*-C_15_H_31_	8.00	1.85	8.47	1.66
PAA-**6**	*n*-C_17_H_35_	7.58	1.85	8.10	1.66

^1^ Mobile phase: 20% isopropyl alcohol in hexane. Flow rate: 1.0 mL/min. Detection: 254 nm UV. *k*_1_: Retention factor for the first eluted enantiomer. In parentheses, the absolute configuration of the first eluted enantiomer is presented. α: Separation factor.

**Table 2:** Resolution of AAEs shown in Figure 2 on CSP **1** and CSP **2** ^1^.

AAEs	R_1_	R_2_	CSP 1		CSP 2	
			*k*_1_	α	*k*_1_	α
AAE-**1**	CH_3_	CH_3_	10.60 (S)	1.93	12.70 (R)	1.55
AAE-**2**	CH_3_	C_2_H_5_	7.47 (S)	1.94	8.70 (R)	1.59
AAE-**3**	(CH_3_)_2_CH	CH_3_	8.70 (S)	2.32	7.44 (R)	1.89
AAE-**4**	(CH_3_)_2_CH	C_2_H_5_	6.50 (S)	2.37	5.57 (R)	1.98
AAE-**5**	C_6_H_5_	CH_3_	27.95 (S)	1.16	30.29 (R)	1.11
AAE-**6**	C_6_H_5_	C_3_H_7_	16.73 (S)	1.23	17.96 (R)	1.19
AAE-**7**	C_6_H_5_	C_4_H_9_	14.49 (S)	1.24	15.36 (R)	1.20
AAE-**8**	C_6_H_5_	C_8_H_17_	10.12 (S)	1.27	12.06 (R)	1.22
AAE-**9**	C_6_H_5_CH_2_	CH_3_	19.78 (S)	1.50	16.10 (R)	1.40
AAE-**10**	C_6_H_5_CH_2_	C_2_H_5_	14.75 (S)	1.57	11.94 (R)	1.47
AAE-**11**	4-HOC_6_H_5_CH_2_	C_2_H_5_	42.36 (S)	1.70	35.93 (R)	1.59

^1^ Mobile phase: 20% isopropyl alcohol in hexane. Flow rate: 1.0 mL/min. Detection: 254 nm UV. *k*_1_: Retention factor for the first eluted enantiomer. In parentheses, the absolute configuration of the first eluted enantiomer is presented. α: Separation factor.

**Table 3:** Resolution of NPAs shown in Figure 2 on CSP **1** and CSP **2** ^1^.

NPAs	R_1_	CSP 1		CSP 2	
		*k*_1_	α	*k*_1_	α
NPA-**1**	CH_3_	5.03 (S)	1.72	5.31 (R)	1.49
NPA-**2**	(CH_3_)_2_CH	2.75 (S)	2.11	2.56 (R)	1.71
NPA-**3**	(CH_3_)_2_CH_2_CH	2.87 (S)	1.79	2.62 (R)	1.64
NPA-**4**	C_6_H_5_	8.92 (S)	1.12	8.71 (R)	1.08
NPA-**5**	C_6_H_5_CH_2_	6.33 (S)	1.62	5.13 (R)	1.47
NPA-**6**	4-HOC_6_H_5_CH_2_	18.85 (S)	1.69	17.49 (R)	1.47

^1^ Mobile phase: 20% isopropyl alcohol in hexane. Flow rate: 1.0 mL/min. Detection: 254 nm UV. *k*_1_: Retention factor for the first eluted enantiomer. In parentheses, the absolute configuration of the first eluted enantiomer is presented. α: Separation factor.

**Table 4:** Resolution of NNDAs shown in Figure 2 on CSP **1** and CSP **2** ^1^.

NNDAs	R_1_	R_2_	CSP 1		CSP 2	
			*k*_1_	α	*k*_1_	α
NNDA-**1**	CH_3_	CH_3_	8.72 (S)	1.53	7.20 (R)	1.32
NNDA-**2**	CH_3_	CH_2_CH_3_	3.32 (S)	1.61	3.29 (R)	1.41
NNDA-**3**	CH_3_	CH_2_CH_2_CH_3_	2.03 (S)	1.58	1.98 (R)	1.41
NNDA-**4**	(CH_3_)_2_CH	CH_3_	3.43 (S)	1.77	2.97 (R)	1.51
NNDA-**5**	(CH_3_)_2_CH	CH_2_CH_3_	2.43 (S)	1.84	2.01 (R)	1.66
NNDA-**6**	(CH_3_)_2_CH	CH_2_CH_2_CH_3_	1.42 (S)	1.74	1.22 (R)	1.61
NNDA-**7**	(CH_3_)_2_CH_2_CH	CH_3_	3.74 (S)	1.48	3.18 (R)	1.40
NNDA-**8**	(CH_3_)_2_CH_2_CH	CH_2_CH_3_	2.21 (S)	1.62	1.90 (R)	1.51
NNDA-**9**	(CH_3_)_2_CH_2_CH	CH_2_CH_2_CH_3_	1.98 (S)	1.69	1.85 (R)	1.62
NNDA-**10**	C_6_H_5_	CH_3_	8.88 (S)	1.22	8.02 (R)	1.20
NNDA-**11**	C_6_H_5_	CH_2_CH_3_	5.80 (S)	1.18	4.76 (R)	1.16
NNDA-**12**	C_6_H_5_	CH_2_CH_2_CH_3_	5.41	1.00	5.36	1.00
NNDA-**13**	C_6_H_5_CH_2_	CH_3_	6.40 (S)	1.53	5.06 (R)	1.36
NNDA-**14**	C_6_H_5_CH_2_	CH_2_CH_3_	3.96 (S)	1.87	3.03 (R)	1.51
NNDA-**15**	C_6_H_5_CH_2_	CH_2_CH_2_CH_3_	3.63 (S)	1.82	3.12 (R)	1.64
NNDA-**16**	4-HOC_6_H_5_CH_2_	CH_3_	15.40 (S)	1.59	12.68 (R)	1.34
NNDA-**17**	4-HOC_6_H_5_CH_2_	CH_2_CH_3_	12.01 (S)	1.93	11.02 (R)	1.49
NNDA-**18**	4-HOC_6_H_5_CH_2_	CH_2_CH_2_CH_3_	10.89 (S)	1.98	10.42 (R)	1.62

^1^ Mobile phase: 20% isopropyl alcohol in hexane. Flow rate: 1.0 mL/min. Detection: 254 nm UV. *k*_1_: Retention factor for the first eluted enantiomer. In parentheses, the absolute configuration of the first eluted enantiomer is presented. α: Separation factor.

**Table 5:** Comparison of the chromatographic results for the resolution of some selected analytes on CSP **1**, CSP **2** and CSP **3** ^1^.

Analytes	CSP 1		CSP 2		CSP 3	
	*k*_1_	α	*k*_1_	α	*k*_1_	α
PPA-**1**	15.98 (S)	2.19	8.53 (R)	1.98	1.90 (S)	2.08
AAE-**1**	10.60 (S)	1.93	12.70 (R)	1.55	2.72 (S)	1.76
AAE-**3**	8.70 (S)	2.32	7.44 (R)	1.89	1.61 (S)	2.14
AAE-**11**	14.75 (S)	1.57	11.94 (R)	1.47	4.36 (S)	1.54
NPA-**1**	5.03 (S)	1.72	5.31 (R)	1.49	1.37 (S)	1.51
NPA-**2**	2.75 (S)	2.11	2.56 (R)	1.71	0.80 (S)	1.79
NPA-**3**	2.87 (S)	1.79	2.62 (R)	1.64	0.65 (S)	1.51
NNDA-**2**	3.32 (S)	1.61	3.29 (R)	1.41	1.68 (S)	1.58
NNDA-**5**	2.43 (S)	1.84	2.01 (R)	1.66	0.80 (S)	1.80
NNDA-**8**	2.21 (S)	1.62	1.90 (R)	1.51	0.78 (S)	1.53
NNDA-**11**	5.80 (S)	1.18	4.76 (R)	1.16	1.40 (S)	1.27
NNDA-**14**	3.96 (S)	1.87	3.03 (R)	1.51	1.10 (S)	1.53
NNDA-**17**	12.01 (S)	1.93	11.02 (R)	1.49	2.46 (S)	1.47

^1^ Mobile phase: 20% isopropyl alcohol in hexane. Flow rate: 1.0 mL/min. Detection: 254 nm UV. *k*_1_: Retention factor for the first eluted enantiomer. In parentheses, the absolute configuration of the first eluted enantiomer is presented. α: Separation factor.

## Abbreviations

The following abbreviations are used in this manuscript:

CSP: Chiral stationary phase
EEDQ: 2-Ethoxy-1-ethoxycarbonyl-1,2-dihydroquinoline
PAA: *N*-(3,5-Dinitrobenzoyl)-1-phenylalkylamines
AAE: *N*-(3,5-Dinitrobenzoyl)-α-amino acid esters
NPA: *N*-(3,5-Dinitrobenzoyl)-α-amino acid *N*-propyl amides
NNDA: *N*-(3,5-Dinitrobenzoyl)-α-amino acid *N*,*N*-dialkyl amides
